# Liver Transplantation for Hepatocellular Carcinoma: Kent Hospital Experience

**DOI:** 10.1007/s12029-017-9964-3

**Published:** 2017-08-04

**Authors:** Cahit Yılmaz

**Affiliations:** Kent Hospital, Sk No:56 Cigli, 8229 Izmir, Turkey

## Aim

This study was conducted to analyze the outcome of cirrhotic patients accompanied with hepatocellular carcinoma (HCC) who underwent liver transplantation (LT) at our institution.

## Patients and Methods

A total of 920 LTs were performed from July 2009 to December 2016. Two hundred sixty-five pediatric recipients were excluded and 655 adult recipients were studied. One hundred seventy-seven (27%) of the adult recipients had HCC in the histopathological examination. Thirty-two (18%) of the patients were transplanted from deceased donors while 145 (82%) were transplanted from live donors. The median age of the patients was 56 (range 18–72) and the median MELD score was 11 (range 6–33). Thirty-eight of the cases (6%) had incidental tumors identified after LT. None of the patients had extrahepatic disease or lymph node metastases. Preoperative radiofrequency ablation or chemoembolization were used in 46 (26%) patients and 6 patients had preoperative hepatic resection. The etiologies were HBV infection (*n* = 105), HCV infection (*n* = 33), cryptogenic cirrhosis (*n* = 16), alcohol (*n* = 15), autoimmune disease (*n* = 2), HCV+HBV (*n* = 2), PBS (*n* = 1), PSC (*n* = 1), tyrosinemia (*n* = 1), and non-alcoholic steatohepatitis (*n* = 1). In 100 (56%) patients, HCC presented as a solitary nodule. Thirty-three patients (19%) had two, 11 patients (6%) had 3, and 33 patients (19%) had 4 or more HCCs. Histopathological examination of the tumors revealed well-differentiated histology in 64 patients (36%), moderate differentiation in 90 patients (51%), and poor differentiation in 23 patients (13%). The median AFP level was 9 (range 1 and >1210). One hundred four of the patients (59%) were within Milan criteria while 73 (41%) of the patients were beyond Milan criteria.

## Results

Median follow-up was 37 months (range 3–89) after LT. One and two-year survival rates for patients were 88 and 82%, respectively. Thirty-three of the HCC patients (18.6%) had recurrence of HCC. The recurrence rate was 10/104 (10%) and 23/73 (32%) for the within Milan and beyond Milan groups, respectively (*p* = .000232). The recurrence rate was 26/145 (18%) and 7/32 (22%) for the live donor and deceased donor groups, respectively (*p* = .616574).

## Conclusion

Outcome of the HCC patients without extra-hepatic invasion who underwent LT is quite well including the patients with large tumors. Transplantation using living donors can help to overcome the scarcity of organs with comparable results to the transplants from deceased donors. Although the recurrence rate is higher for the patients beyond Milan criteria, LT can provide 68% disease-free survival for this group of patients.

